# Chronische Pankreatitis: Charakterisierung und Differenzierung zum Pankreaskarzinom

**DOI:** 10.1007/s00117-021-00857-9

**Published:** 2021-05-17

**Authors:** Antonia Kristic, N. Bastati, S. Poetter-Lang, A. Messner, A. Herold, D. Tamandl, Ahmed Ba-Ssalamah

**Affiliations:** Department of Biomedical Imaging and Image-guided Therapy, Medical University of Vienna, General Hospital of Vienna (AKH), Waehringer Guertel 18–20, 1090 Wien, Österreich

**Keywords:** Entzündung, Benigne Pankreasläsionen, Maligne Pankreasläsionen, Computertomographie, Magnetresonanztomographie, Inflammation, Benign pancreatic lesions, Malign pancreatic lesions, Computed tomography, Magnetic resonance imaging

## Abstract

**Klinisches/methodisches Problem:**

Bei der chronischen Pankreatitis (CP) handelt es sich um eine langanhaltende Entzündung der Bauchspeicheldrüse, welche die normale Struktur und Funktion des Organs schädigt. Das breite Spektrum an entzündlichen Pankreaserkrankungen umfasst einzelne Entitäten, wie die fokale Pankreatitis (FP) oder den Pseudotumor („mass-forming pancreatitis“), welche radiomorphologisch ein Adenokarzinom der Bauchspeicheldrüse (PDAC) nachahmen können. In weiterer Folge kann eine Fehldiagnose zu einem vermeidbaren und unnötigen operativen Eingriff oder zu einer Therapieverzögerung führen.

**Radiologische Standardverfahren:**

Der Ultraschall (US) ist das primäre bildgebende Verfahren zur Abklärung von Pankreaserkrankungen, gefolgt von kontrastmittelverstärkter Computertomographie (KM-CT), die als meistverwendete Methode bei der diagnostischen Abklärung von Bauchspeicheldrüsenerkrankungen gilt. Die Magnetresonanztomographie (MRT) und/oder die MR-Cholangiopankreatographie (MRCP) können als Problemlöser eingesetzt werden, um zwischen soliden und zystischen Läsionen zu unterscheiden sowie auch Anomalien der Pankreasgänge auszuschließen, welche bei rezidivierender akuter Pankreatitis (AP) vorhanden sein können, oder um frühe Anzeichen einer CP zu visualisieren. Die MRCP hat dabei die diagnostische endoskopische retrograde Cholangiopankreatographie (ERCP) in der Abklärung von therapeutischen Interventionen im Wesentlichen ersetzt.

**Empfehlung für die Praxis:**

Folgender Übersichtsartikel fasst die relevanten Merkmale in der Computertomographie (CT) und MRT zusammen, um eine akkurate, frühzeitige Diagnose einer CP zu stellen und eine Differenzierung zwischen FP und Pankreaskarzinom zu ermöglichen, um somit – auch in schwierigen Fällen – ein adäquates Therapiemanagement zu gewährleisten.

## Abstract (en)

**Clinical/methodological issue:**

Chronic pancreatitis (CP) is a long-lasting inflammation of the pancreas that changes the normal structure and function of the organ. There are a wide range of inflammatory pancreatic diseases, of which some entities, such as focal pancreatitis (FP) or “mass-forming pancreatitis,” can mimic pancreatic ductal adenocarcinoma (PDAC). As a consequence, a misdiagnosis can lead to avoidable and unnecessary surgery or delay of therapy.

**Standard radiological methods:**

The initial imaging method used in pancreatic diseases is ultrasound due to its availability and low cost, followed by contrast-enhanced computed tomography (CE-CT), which is considered a workhorse in the diagnostic work-up of diseases of the pancreas. Magnetic resonance imaging (MRI) and/or MR cholangiopancreatography (MRCP) can be used as a problem-solving tool to distinguish between solid and cystic lesions, and to rule out abnormalities in the pancreatic ducts, such as those associated with recurrent acute pancreatitis (AP) or to show early signs of CP. MRCP has essentially replaced diagnostic endoscopic retrograde cholangiopancreatography (ERCP) in the initial assessment before any therapeutic intervention.

**Practical recommendation:**

The following review article summarizes the relevant features of CT and MRI that can help to make the diagnosis of CP and to aid in the differentiation between focal pancreatitis and PDAC, even in difficult cases.

Die chronische Pankreatitis ist eine lang andauernde Entzündung mit unterschiedlichen morphologischen Erscheinungsformen. Hierzu zählt auch die fokale Pankreatitis („mass-forming pancreatitis“), die eine diagnostische Herausforderung in der Differenzierung zum Pankreaskarzinom darstellen kann, da sich bildmorphologische sowie klinische Parameter überschneiden können. Eine akkurate und frühzeitige Diagnosestellung ist daher essenziell, um eine adäquate Therapie zu ermöglichen.

Die chronische Pankreatitis (CP) entwickelt sich bei ca. 4–24 % der Patienten aus rezidivierenden Schüben einer akuten Pankreatitis (AP), kann aber ebenso durch eine primär chronische, progrediente Entzündung der Bauchspeicheldrüse verursacht werden. Dieses Krankheitsbild führt zu einer irreversiblen Fibrose mit Atrophie und in weiterer Folge zu einer endokrinen und exokrinen Funktionsstörung des Drüsenorgans [24]. Radiologisch bereitet die Diagnosestellung einer fortgeschrittenen CP keine Schwierigkeiten, da die typischen Merkmale wie Kaliber- und/oder Konturunregelmäßigkeiten, Stenosen und Dilatationen des Pankreashauptgangs und seiner Nebenäste, Verkalkungen (parenchymatös viel häufiger als intraduktal) sowie eine Atrophie des Organs gut erkennbar sind [14].

Die CP erhöht das Risiko für die Entwicklung eines Pankreaskarzinoms, laut einer Metaanalyse, um das 6‑ bis 12-Fache, bei Patienten mit hereditärer Pankreatitis sogar um das 70-Fache im Vergleich zur Allgemeinbevölkerung [2]. Zudem stellt eine Unterscheidung zu einer umschriebenen Raumforderung bzw. Pseudotumor im Rahmen einer CP mittels Schnittbildverfahren oftmals eine große Herausforderung dar, weshalb in solchen Fällen differenzialdiagnostisch immer auch das Pankreaskarzinom in Betracht gezogen werden sollte, bis die endgültige Diagnose durch weitere Untersuchungen belegt ist.

## Bildgebende Merkmale der Pankreatitis

Der transabdominelle Ultraschall (US) stellt eine kosteneffektive und schnell verfügbare Methode dar, welche oft zur Erstdiagnostik von Pankreaserkrankungen herangezogen wird. Jedoch verzögert dieser Schritt oftmals die Abklärung. Das Pankreas kann aufgrund von Gasüberlagerungen etc. häufig nicht suffizient beurteilt werden, sodass anschließend meist eine Schnittbilddiagnostik notwendig ist [5, 25].

Die Multidetektor-Computertomographie (MDCT) stellt die primäre Bildgebungsmodalität für die Beurteilung und Diagnosestellung der Pankreaserkrankungen im Allgemeinen und der CP im Speziellen dar. Mittels CT können typische Veränderungen der CP einfach erkannt werden [5]. Aufgrund der aktiven Entzündung erscheint die Bauchspeicheldrüse im Frühstadium der CP eher vergrößert und geschwollen, während im fortgeschrittenen Stadium häufig Verkalkungen und die Organatrophie auftreten [5].

Die Magnetresonanztomographie (MRT) ist zur Visualisierung subtiler Befunde im Rahmen einer frühen CP, einschließlich beginnender Gangunregelmäßigkeiten, Verlust der typischen Organläppchenstruktur sowie Signalintensitätsänderungen des Pankreasgewebes, der MDCT überlegen [5].

Darüber hinaus haben sich neben der konventionellen MRT sowohl diffusionsgewichtete Sequenzen als auch T1-Mapping-Techniken als vielversprechend erwiesen [16]. Daher ist die MRT, und insbesondere die Sekretin-verstärkte MR-Cholangiopankreatographie (MRCP), bei der Diagnose einer frühen CP inklusive der exokrinen Funktionsbeurteilung sehr hilfreich [4].

Eine rezente Metaanalyse inkludierte 43 Studien und untersuchte die diagnostische Genauigkeit zur Detektion einer CP. Die beschriebenen summierten Sensitivitäten betrugen 67 %, 75 %, 78 %, 81 % und 82 % für US, CT, MRCP, endoskopischen Ultraschall (EUS) und die endoskopische retrograde Cholangiopankreatographie (ERCP). Die Spezifitäten lagen bei allen Modalitäten über 90 % [10].

Die gefürchtetste Komplikation einer CP ist die Entstehung eines Pankreaskarzinoms [18]. Das radiologische Bild eines inflammatorischen Pseudotumors („mass-forming pancreatitis“), vor dem Hintergrund des erwähnten erhöhten Pankreaskarzinomrisikos bei CP, stellt vor allem bei den 3 Sonderformen der Erkrankung, nämlich der fokalen Pankreatitis (FP), Groove-Pankreatitis (GP) und Autoimmunpankreatitis (AIP) häufig eine Herausforderung in der Differenzierung dieser Entitäten von einem Pankreaskarzinom dar.

Es wird geschätzt, dass bei etwa einem Drittel aller Patienten, welche von diesen 3 Formen, insbesondere der GP, betroffen sind, die Pankreasresektion aufgrund eines Karzinomverdachts vermeidbar gewesen wäre [26]. Da bildgebende sowie klinische Parameter oft überlappend und in manchen Fällen sogar wiederholte Biopsie-Ergebnisse nicht aussagekräftig bzw. unklar sind [17], sollen hier weitere sekundäre bildgebende Merkmale diskutiert werden, die zwischen entzündlichen und malignen Entitäten differenzieren können [26, 28]. Die genaue Kenntnis dieser sekundären Zeichen kann dem Radiologen bei der Unterscheidung CP vs. Pankreaskarzinom helfen und auch die Malignitätswahrscheinlichkeit abschätzen.

Deshalb werden wir gesondert auf die drei entzündlichen Sonderformen der CP eingehen, die im Speziellen oft Schwierigkeiten bei der Differenzierung zu einem Pankreaskarzinom bereiten.

### Fokale Pankreatitis

Die fokale Pankreatitis (FP) kann sich in bis zu 50 % aller CP-Fälle als umschriebener raumfordernder Prozess darstellen. Sowohl die FP als auch das Pankreaskarzinom kommen größtenteils in der kontrastmittelverstärkten Computertomographie (KM-CT) hypo- oder isodens zur Darstellung, sind meist im Pankreaskopf lokalisiert und führen oft zu einer konsekutiven Erweiterung des Pankreashauptgangs. Die Genauigkeit der CT zur Differenzierung zum Pankreaskarzinom beträgt daher nur 77 %. Auch in der Gadolinium-verstärkten, dynamischen MRT ist die FP in der arteriellen und venösen Phase hypointens, und in der späten Phase hypo- bis isointens. Das dynamische Kontrastmittelverhalten kann bei der FP und dem Pankreaskarzinom sehr ähnlich sein. Es gibt jedoch bildgebende Merkmale, die helfen, die beiden Entitäten voneinander zu unterscheiden [26]. Während es beim Pankreaskarzinom zu einer Infiltration der peripankreatischen Gefäße kommt, zeigen sich bei der FP allenfalls Gefäßkaliberveränderungen im Rahmen einer Kompression oder Verlagerung [22, 26]. Die Gefäßinfiltration der peripankreatischen Arterien ist spezifisch und deutet auf ein bereits lokal fortgeschrittenes Pankreaskarzinom hin.

Eine Verlagerung bzw. Verdrängung der parenchymalen Verkalkungen in Zusammenhang mit einer neu aufgetretenen fokalen Weichteilmasse bei einer diffus verkalkten CP sollte den Verdacht auf ein Pankreaskarzinom bei CP erhärten [23].

**Figure 1:**
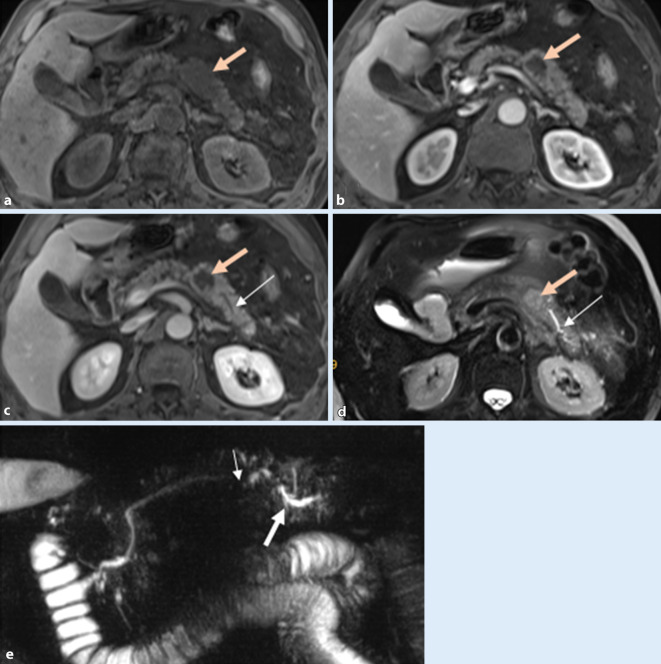

**Figure 2:**
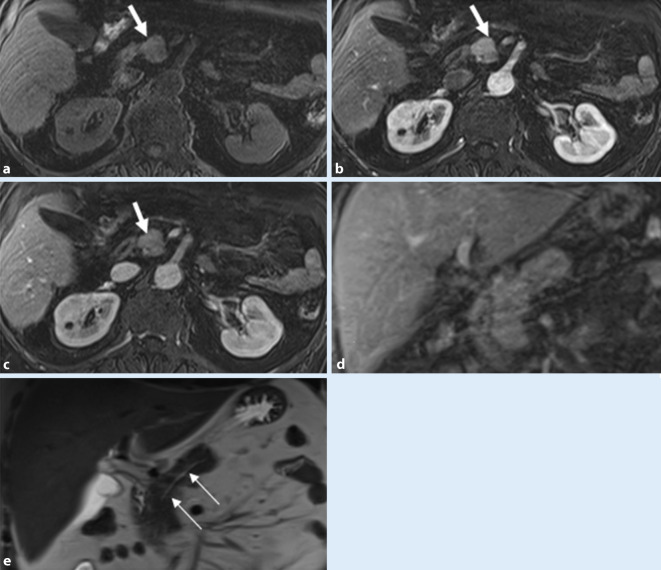

Morphologische Pankreasgangveränderungen im Rahmen eines Pankreaskarzinoms oder einer entzündlichen Striktur bei CP können sehr unterschiedlich sein. Im fortgeschrittenen CP-Stadium zeigen sich neben fokalen, segmentalen Strikturen und dilatierten und deformierten Seitenästen auch Konturunregelmäßigkeiten des Hauptgangs. Die Dilatation des Pankreasgangs distal des Pankreaskarzinoms ist in der Regel ausgeprägt (Abb. 1), jedoch glatt begrenzt und mit einer deutlichen Parenchymatrophie assoziiert [26]. Eine glatte Verengung des Pankreasgangs, welcher die Raumforderung ohne eine abrupte oder vollständige Obstruktion durchquert, das „duct-penetrating sign“, spricht für die Diagnose einer FP (Abb. 2). Die zusätzliche Durchführung einer Sekretin-verstärkten MRCP, falls verfügbar, kann zur besseren Veranschaulichung dieses Zeichens sehr hilfreich sein ([4]; Tab. 1).

**Table 1:** 

Bildparameter	Pankreatitis	Pankreaskarzinom	Kommentare
„Duct-penetrating sign“	Möglich	Fehlend	Verlässliches Zeichen (Spezifität 96 %, Sensitivität 85 %) [9]
„Double duct sign“	Fehlend	Möglich	Spezifität 63–80 %, Sensitivität 50–76 % [21]
Abrupte Hauptgangokklusion	Fehlend	Möglich	Malignität bei 58 % [11]
Multiple segmentale Stenosen	Möglich	Fehlend	Bei 46,1 % einfache und bei 53,8 % multiple Hauptgangstenosen [20]
Verlagerung von Verkalkungen	Fehlend	Möglich	Keine genauen Angaben
Gefäßinfiltration	Fehlend	Verlässliches Zeichen	Sensitivität 100 % [27]
Gefäßkompression	Möglich	Möglich	Sensitivität 50 % [27]
Kontrastmittelverhalten	Iso- oder leicht hyperintens/hyperdens	Persistent hypointens/hypodens	Sensitivität 93,8 % und Spezifität 66,7 % [28]
Diffusion: ADC-Wert	Höher 1,47 ± 0,18	Niedriger 1,17 ± 0,23	Sensitivität des ADC-Werts 84,4 % [28]
PET-CT	Schwache Tracer- Aufnahme	Starke Tracer-Aufnahme	Sensitivität 92 % und Spezifität 85 % [6]

*PET-CT *Positronen-Emissions-Tomographie*, ADC *„apparent diffusion coefficient“

Falls eine nichtinvasive radiologische Diagnose nicht möglich ist, wird zur weiteren Abklärung meist eine EUS-gezielte Feinnadelbiopsie durchgeführt. Diese ist jedoch oftmals inkonklusiv und daher für die Diagnostik bzw. Therapie-Entscheidung nicht immer hilfreich [26]. Insbesondere bleibt zu erwähnen, dass die diagnostische Sicherheit der EUS bei Vorliegen einer CP auf Sensitivitäten unter 75 % herabfällt [8]. Bei nicht eindeutigen Ergebnissen wird daher in bestimmten Fällen eine operative Exploration oder eine Resektion durchgeführt.

### Groove-Pankreatitis

Die GP ist auch als paraduodenale Pankreatitis oder zystische Duodenaldystrophie bekannt und stellt eine fokale Form der CP dar, die mit Alkoholabusus assoziiert ist. Lokalisiert ist sie in der *Furche* zwischen Zwölffingerdarm, Bauchspeicheldrüsenkopf und Hauptgallengang [1, 26].

**Figure 3:**
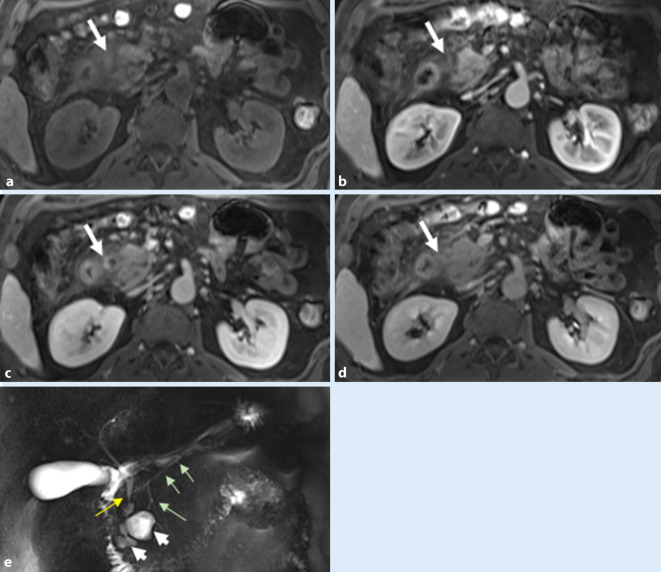

Man unterscheidet zwei Varianten der GP: eine *reine* Form, welche lediglich die *Rinne* zwischen Duodenum, Hauptgallengang und Pankreaskopf betrifft, und die segmentale Form (Abb. 3), bei der sich die fibrösen Strukturveränderungen auch auf den Pankreaskopf ausbreiten [1]. Diese entzündlichen Veränderungen können zur Auftreibung und Strukturalteration des Pankreaskopfes im Sinne eines Pseudotumors führen und ein Karzinom nachahmen, zudem durch fibrotische Veränderungen auch ein „double duct sign“ sichtbar sein [26].

Die klassischen MDCT-Bildgebungsmerkmale (bei der reinen Form der GP) bestehen aus dem Verlust von Fettschichten zwischen dem Pankreaskopf und dem Duodenum mit einer schlecht definierten, sichelförmigen, offenen Weichteilmasse. Das Weichteilgewebe erscheint als *blattartige*, gekrümmte Sichelform, die auf koronalen, multiplanaren Reformationen besser beurteilt werden kann.

Die frühe Phase der kontrastmittelverstärkten dynamischen CT zeigt ein isodenses Areal, bedingt durch eine breite Fibrose. In der Spätphase der dynamischen CT zeigt das fibrotische Gewebe eine verzögerte Anreicherung. Dieses KM-Enhancement ist ein wichtiges Merkmal für die Differenzierung zum Pankreaskarzinom, welches hypodens imponiert.

Auch in der MRT-Bildgebung zeigt sich die GP als blattförmige Masse zwischen Pankreaskopf und Duodenum, welche auf T1-gewichteten Bildern hypointens und je nach Zeitpunkt des Krankheitsbeginns auf T2-gewichteten Bildern hypo-, iso- oder leicht hyperintens ist, mit den typischen Zysten in der Duodenalwand. Vergleichbar zur CT zeigt die fibrotische Masse in den Gadolinium-verstärkten T1-gewichteten Sequenzen im Gegensatz zum Pankreaskarzinom eine verzögerte Anreicherung.

Mittels MRCP sind die Zysten entlang der Duodenalwand besser visualisierbar, und die glatte Kompression des distalen Hauptgallengangs ist gut beurteilbar (Abb. 3).

Im Rahmen einer segmentalen GP kann die MRCP auch eine Dilatation des Pankreashauptgangs oder ein „double duct sign“ aufzeigen, wohingegen diese bei der reinen Form meist nicht erweitert sind [1].

### Autoimmunpankreatitis

Die AIP macht 2–10 % der gesamten CP-Fälle aus [3]. Obwohl der Entstehungsmechanismus nicht gänzlich geklärt ist, werden immunologische und genetische Faktoren als ursächlich vermutet [13].

Die AIP wird in zwei verschiedene Subgruppen einteilt, die sich voneinander durch pathologische und klinische Merkmale unterscheiden [12]. Gemeinsame Merkmale sind das Fehlen erhöhter Entzündungsparameter und Fieber im klinischen Kontext [19] sowie das gute Ansprechen der Patienten auf Kortikosteroide, welche sowohl therapeutisch als auch diagnostisch eingesetzt werden [15].

Histologisch kennzeichnet sich die Typ-1-AIP-IgG4-Subgruppe durch eine lymphoplasmozytäre Infiltration und Fibrose des Organs. Im Gegensatz dazu weist die Typ-2-AIP histologisch neutrophile Infiltrate und epitheloide Zellgranulome auf und zeigt laborchemisch keinerlei Serum-IgG4-Erhöhung [7].

**Figure 4:**
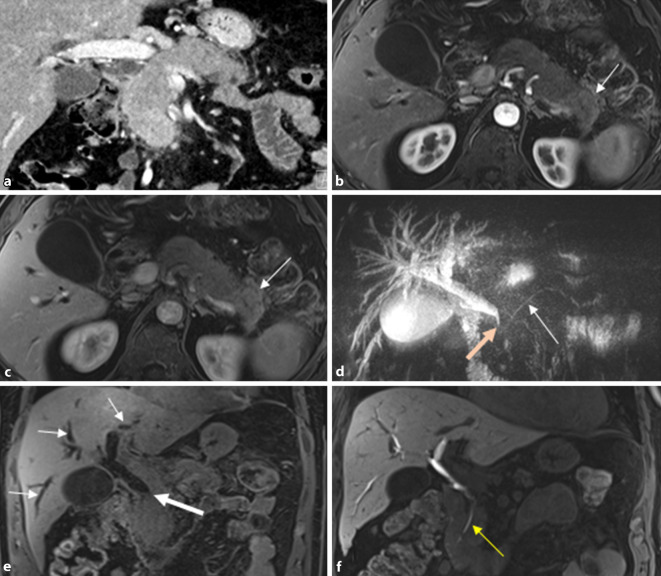

Mittels Schnittbildverfahren kommt oft zumindest eine der drei typischen Manifestationsformen zur Darstellung:

- eine diffuse *wurstartige* Auftreibung (Abb. 4),
- eine fokale Schwellung (30–40 %), meist auf den Pankreaskopf begrenzt, oder
- eine multifokale Pankreasbeteiligung [23].

**Figure 5:**
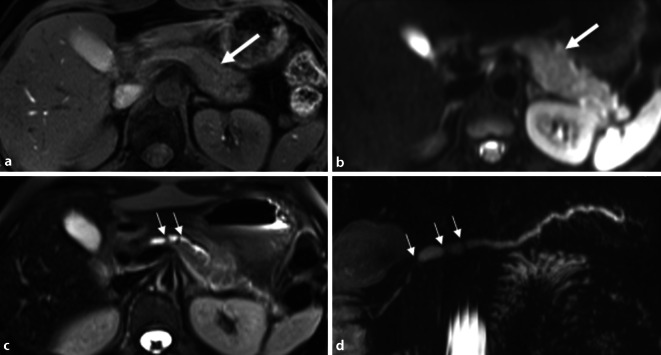

Die ausgeprägte Dilatation des Pankreashauptgangs sowie auch die Organatrophie fehlen bei der AIP typischerweise. Eine markante Gangdilatation oder abrupte Lumeneinengung weisen auf eine maligne Obstruktion hin, wohingegen langstreckige Einengungen des Hauptgangs oder multiple, segmentale Stenosen für eine fokale Entzündungsreaktion sprechen ([13, 17]; Abb. 5). In schwierigen Fällen können die diffusionsgewichteten Sequenzen (DWI) der MRT helfen, die AIP mit signifikant höheren ADC-Werten (Tab. 1) im Pseudotumor von einem Pankreaskarzinom zu unterscheiden [28].

Zur Differenzierung zwischen Pankreaskarzinom und AIP kann die F18-Fluordesoxyglukose(FDG)-Positronen-Emissions-Tomographie (PET) hilfreich sein. Dabei wurde eine Sensitivität von 92 % und eine Spezifität von 85 % berichtet [6]. Zusätzlich liefert die Messung des „standard uptake value“ (SUV) ebenso Hinweise zur Differenzialdiagnose, da die fokale AIP geringere SUV-Werte als das Pankreaskarzinom aufweist [23].

## Fazit für die Praxis

- Die CP kann sich als *typische* Form mit Atrophie und Verkalkungen, als Pseudotumor bzw. Sonderformen i. S. einer FP, GP bzw. AIP manifestieren. Diese Formen können jedoch ein Pankreaskarzinom nachahmen.
- Morphologische Kriterien wie Verkalkungen des Pankreasparenchyms sind bei CP häufig zu sehen, eine Verschiebung der Verkalkungen kann auf ein neues zugrundeliegendes Malignom hinweisen, ebenso wie das „double duct sign“, dieses kann aber in seltenen Fällen auch durch eine entzündliche Erkrankung auftreten.
- Das „duct-penetrating sign“ begünstigt stark die Diagnose einer FP, ebenso wie die Verdrängung (im Gegensatz zur Umscheidung) des Hauptgallengangs und/oder der gastroduodenalen Arterie. Die Visualisierung multipler Pankreas- oder Gallengangstrikturen anstelle einer solitären Striktur und das Fehlen einer substanziellen Dilatation der Seitenäste oder des stromaufwärts gelegenen Pankreasgangs favorisieren ebenso eine FP.
